# Stakeholder perceptions on the impact of trade and investment agreements on nutrition policy space in small island developing states

**DOI:** 10.1186/s12992-024-01091-3

**Published:** 2025-03-03

**Authors:** Noah Bunkley, Judith McCool, Kelly Garton

**Affiliations:** Faculty of Medical and Health Sciences, The University of Auckland, Auckland, New Zealand

## Abstract

**Background:**

Trade liberalisation has contributed to obesogenic food environments globally. Small Island Developing States (SIDS) have some of the world’s highest rates of obesity and nutrition-related noncommunicable diseases. Nutrition regulations have been recognised as necessary population health measures for combating malnutrition, however, legally-binding trade and investment agreements (TIAs) can constrain the policy options available to governments. Geographical, economic, historical, and cultural contexts of SIDS may place them at greater risk of TIA constraints resulting in barriers to the uptake of public health nutrition policies. This article explores the perceptions and experiences of key SIDS nutrition and trade policy stakeholders regarding SIDS’ ability to formulate and implement healthy nutrition policies in the context of TIAs.

**Methods:**

Twelve semi-structured interviews were conducted with key Pacific and Caribbean stakeholders. Analysis was performed via a critical realist grounded theory approach. TIA constraints to policy space, challenges faced by SIDS, and solutions for improving nutrition policy space were identified.

**Findings:**

Participants identified that TIAs did not substantively constrain nutrition policy so long as the policy targeted a legitimate public health objective, was evidenced-based, non-discriminatory, non-arbitrary, necessary, and the least trade-restrictive measure available. However, TIAs were perceived to pose structural and procedural constraints in the form of regulatory chill, increased burden of ensuring trade-compliant nutrition policies, unfair TIA negotiation processes, inconsistent perceptions of ‘unhealthy’ foods, trade liberalisation ideology, and industry interference. These constraints were noted to be particularly acute for SIDS due to their financial and capacity constraints, industry influence and limited international power.

**Conclusion:**

TIA obligations were deemed unlikely to substantively prevent meaningful public health nutrition policies from being developed and implemented in SIDS if nutrition policy met specific trade principles. However, concerns were noted that some of these principles may impose procedural and structural constraints that risked preventing, postponing or diluting potential nutrition policies. These constraints may be particularly problematic for SIDS due to their contextual challenges. Despite this, local, regional and international actors can increase SIDS’ policy space through capacity building, fostering multisectoral collaboration, developing conflict of interest policies, improving TIA negotiation processes, and championing the prioritisation of public health nutrition in trade governance.

## Background

Over the last several decades, the world has been subject to an increasingly obesogenic global food environment shaped by globalisation, neoliberalism, and trade liberalisation [1, 2]. What has been coined the ‘neoliberal diet’ consists of low-cost, high-calorie, low-nutritional value foods that favour taste and cost-saving over nutrition and are often high in fat, salt and sugar [3]. Trade liberalisation—characterised by the proliferation of international trade and investment agreements (TIAs), which promote reducing barriers to imports and exports, harmonisation of regulations, and measures to encourage foreign direct investment and privatisation of state assets—has facilitated the rapid spread of the ‘neoliberal diet’ across the globe [1, 3–9].

While TIAs have been designed to facilitate international trade and investment, their scope has gradually increased, reaching within the domestic sphere to influence domestic policy in the name of regulatory harmonisation, investment standards, protecting intellectual property, and ensuring health and safety, and where states fail to meet their TIA obligations, they may face state-state or investor-state disputes/challenges [10]. Within TIAs, binding rules and principles are set to prevent hidden protectionist policies masquerading as legitimate regulations. Therefore, to be permitted within the international trade and investment regime, domestic policies must not be discriminatory, arbitrary, or perceived to constitute unfair treatment to any trading partner (or investor) and be the least trade-restrictive option to achieve the policy objective, else they are construed as hidden protectionism [11].

For nutrition policy, the interference of trade interests in domestic regulatory autonomy can reduce the ‘policy space’ governments have for developing and implementing healthy nutrition policies where regulations intersect with binding trade commitments. Policy space is defined as “the freedom and ability of a government to identify and pursue the most appropriate mix of economic and social policies to achieve equitable and sustainable development that is best suited to its particular national context” [12]. Critically, TIA obligations can have a dampening effect on nutrition policy development, known as regulatory chill, where TIAs can result in inaction on public policy due to the threat of state-state or investor-state dispute challenges [13–16]. This is especially pertinent for small island developing states (SIDS), which may have limited capacity to formulate and administer trade policy and have limited representation in international trade forums, making it difficult for them to adhere to—and have their say in—WTO and other trade and investment governing bodies’ procedures and rules [17].

The modern system of TIAs arose in the mid-1990s amidst a resurgence of neoliberalism in Western democracies as a preferred economic system. As a political approach, neoliberalism favours free-market capitalism, and as such is generally associated with economic liberalization policies like privatization, deregulation, globalization, free trade, austerity, and reductions in government spending on public services in order to increase the role of the private sector in the economy and society—and minimize the role of government [18]. Under this policy paradigm, private sector interests are powerful economic operators with significant influence in government decision-making. It has also been argued that this system affords these private sector interests the ability to reframe health narratives, define social norms, influence the creation and use of knowledge, and set the rules that govern commercial interactions [18–21]. Thus, TIAs are only one way in which a neoliberal policy paradigm constrains the development and implementation of healthy public policies.

While this paper acknowledges that trade agreements and international investment agreements are different types of treaties with different sets of rules, they are increasingly intertwined in modern economic arrangements. Though the substantive constraints to policy and effects on food environments may differ between specific agreements, the procedural and structural constraints they pose, as defined by Fidler et al. (22) and by Garton et al. [23], tend to be similar, and these are the focus of this research. Therefore, this research refers to TIAs as an encompassing term. Table 1 represents a shorthand collection of the potential claims that have, or might be raised for different areas of population nutrition policy based on existing TIAs. Readers should note that the table is by no means exhaustive, is an oversimplification of what are complex and context-specific interactions, and does not assess the validity of the legal argument or of any successful defence.

**Table 1:** Areas of intersection and potential conflict between TIAs and population nutrition policies. (adapted from Garton et al. 2021 [24], Garton et al. 2023 [22])

Agreement / Chapter of TIAs	Areas of population nutrition policy potentially affected	Basis of potential arguments against nutrition policy
WTO General Agreement on Tariffs and Trade (GATT)	Fiscal policies: import tax, excise tax (e.g. based on ingredients, nutrient content, degree of processing), product bans	Quantitative restrictions, discrimination between ‘like’ products
WTO Technical Barriers to Trade (TBT) Agreement, and TBT chapters of other TIAs	Nutrition labelling (interpretive, health warnings), marketing restrictions, nutrient limits/bans; any public health nutrition regulations (mandatory) underpinned by regulatory distinctions related to the characteristics of traded goods	Discrimination, necessity/trade restrictiveness, (lack of) scientific evidence
WTO Agreement on Trade-Related Aspects of Intellectual Property Rights (TRIPS), and Intellectual Property (IP) chapters of other TIAs	Marketing restrictions and product labelling (pertaining to brand logos or images that are registered trademarks)	Unjustified restriction on use of trademarks
WTO General Agreement on Trade in Services (GATS), and Services chapters of other TIAs	Retail (e.g. fast food, supermarket) services - e.g. mandatory labelling, shelf space/displays, sales bans (e.g. to minors); advertising services	Restriction on supply of a service
WTO Agreement on Sanitary and Phytosanitary Measures (SPS Agreement), and SPS chapters of other TIAs	Food safety requirements, prohibition of specific harmful ingredients or processes	Discrimination, necessity/trade restrictiveness, (lack of) scientific evidence
Investment treaties, and Investment chapters of other TIAs	Any nutrition policy, if it unfairly affects a foreign investor	Fair and equitable treatment, indirect expropriation
Transparency, Regulatory coherence	Any nutrition policy, if it affects the process of policymaking, e.g. to curb industry interference	Lack of transparency or notification; failure to adhere to good regulatory practice

SIDSs are a group of 58 localities situated in the three geographical regions of the Pacific, the Caribbean, and the Atlantic, Indian Ocean and South China Sea and are characterised by their small size, limited resources, geographical dispersion and global isolation. They have formed a unified group addressing many issues they have collectively faced, such as the rising burden of non-communicable diseases (NCDs) and climate change [24]. Despite their similarities, their populations are culturally diverse, with various languages, traditions, and historical experiences. This paper focuses on the experiences of SIDS in the Caribbean and Pacific in relation to nutrition policy and trade. Six Pacific and 16 Caribbean countries are members of the World Trade Organization (WTO) [25] and there are several regional and bilateral TIAs and customs unions that are active within each region.

Healthy nutrition policies are needed to combat rising NCDs in SIDS in the Pacific and the Caribbean such as those targeting the food environment, including fiscal policies, interpretive nutrition labelling, restrictions on marketing and advertising, and limits on critical nutrients. However, the international trade regime structure, as set within the conglomeration of WTO obligations or through other bilateral or multilateral trade and investment agreements, may result in barriers to developing and implementing healthy nutrition policies in accordance with international best practices and World Health Organization (WHO) recommendations [22, 26]. Despite their theoretical vulnerability to TIA-related nutrition policy space constraints, there are several examples of SIDS moving forward with bold population nutrition regulations, with over three-quarters of Pacific SIDS having implemented a sugar-sweetened beverages (SSB) tax and one (non-WTO member state) banning SSBs outright [27, 28].

This research aimed to explore the perceptions and experiences of key stakeholders operating in the nutrition and trade policy sphere within SIDS in the Pacific and the Caribbean in relation to nutrition policy space and TIAs. The analysis was guided by two main research questions:

1. How do TIAs impact nutrition policy space for SIDS in the Pacific and the Caribbean?

2. What are some potential strategies for expanding nutrition policy space for SIDS in the Pacific and the Caribbean in the context of TIAs?

## Methods

### Theoretical foundations

This study was based on grounded theory methodology within a critical realist paradigm. A critical realist grounded theory interprets pre-existing theoretical knowledge as a point of departure that acts as building blocks for further theory development [29]. The combination of grounded theory and critical realism has previously been applied successfully in other fields [29–31]. Fidler, Aginam and Correa’s framework of the substantive, procedural, and structural constraints posed by TIAs on policy space (22), as further developed by Garton, Swinburn and Thow [22], was used as a starting point to establish the hypothesis of this research. This study further developed the framework whereby ‘Structure’ was taken broadly to include the physical, institutional and ideological constructs that shape nutrition policy decision-making.

### Participant recruitment

Participants were recruited through stratified purposive sampling and selected based on their nutrition policy and trade policy expertise within the Pacific or Caribbean. The starting point for our sampling was our professional network of trade and nutrition policy experts in the Pacific and Caribbean. Further participants were identified by snowballing from recommended contacts of interviewed participants. Given the specific expertise required, four to eight participants in each of the Caribbean and the Pacific were considered likely sufficient to reach thematic saturation at the outset of the research and within feasibility constraints. Saturation was thought to be achieved when no new information was produced from the interviews. Exclusion criteria for participants included people who did not have expertise in trade or food policy, people with expertise in nutrition and trade policy but who did not have expertise in the Pacific or Caribbean and people unable to converse in English.

### Interview design and conduct

One-on-one, semi-structured interviews were conducted via Zoom between December 2021 and February 2022. The interview guide was informed by a literature review [26] and included questions aimed at understanding the experiences of key stakeholders in developing nutrition policies in the context of TIAs and identifying strategies for expanding nutrition policy space. In keeping with grounded theory methodology, the interview guide was adapted over successive interviews in response to the identification of new information and areas requiring further inquiry [31]. The interview guide provided participants with the opportunity to discuss the nutrition policies and TIAs they deemed relevant for developing healthy food environments in their regions. No specific policies or TIAs were mentioned by the interviewer without first being discussed by the participant.

Interviews lasted between 30 and 90 min. The recordings were transcribed verbatim by the lead researcher. Where requested on the consent forms, audio recordings and transcripts were returned to the participants for review and correction.

### Data extraction and synthesis

The first coding stage involved open coding, which used induction and grounded theory principles to be open-minded to new theoretical possibilities arising from the data [31]. The transcripts were initially coded line-by-line in process codes that captured conceptual items with informal noting of first impressions. Where possible, original terms from the interviewee were used. During the open coding process, provisional categories containing multiple codes were created, and coding labels and less relevant codes were refined.

The second stage involved axial coding, in which empirical data was redescribed using theoretical concepts. The process sought to make explicit the connections between concepts and categories and to go beyond empirical data and thick descriptions to identify links between the codes [31]. Coded data was finally grouped into themes that are described in the results.

### Ethics

Ethics approval was obtained through the University of Auckland Human Participants Ethics Committee (UAHPEC23180).

## Results

Eighteen stakeholders were approached via email for interview, twelve of whom responded to and accepted the request (six working in the Caribbean, and six working in the Pacific). Of these, five were academics, four were governmental policy advisors, and three were non-governmental policy advisors, all working in trade and nutrition policy (Table 2). All interviews were conducted online via Zoom. There was a reasonable degree of agreement between participant responses, and we were confident to have reached saturation for an exploratory study.

**Table 2:** List of stakeholder interviewees

Number	Code	Region	Area of work
1	CA1	Caribbean	Academic
2	CNG2	Caribbean	Non-governmental policy advisor
3	CG3	Caribbean	Governmental policy advisor
4	CNG4	Caribbean	Non-governmental policy advisor
5	CG5	Caribbean	Governmental policy advisor
6	CG6	Caribbean	Governmental policy advisor
7	PA1	Pacific	Academic
8	PA2	Pacific	Academic
9	PA3	Pacific	Academic
10	PNG4	Pacific	Non-governmental policy advisor
11	PG5	Pacific	Governmental policy advisor
12	PA6	Pacific	Academic

Themes were identified and grouped within three overarching categories (i) the trade- and investment-related constraints on nutrition policy space, (ii) specific challenges SIDS face in the nutrition policy and trade sphere, and (iii) potential solutions for reducing these constraints to improve nutrition policy space.

### Trade- and investment-related constraints on nutrition policy space

#### Substantive constraints

Substantive constraints refer to limits on types of nutrition policy choices through the express prohibition of policy options (22). Participants largely believed that most healthy nutrition policies were not substantively constrained by TIAs so long as the nutrition policy targeted a legitimate public health objective, was evidenced-based, non-discriminatory, non-arbitrary, necessary, and the least trade-restrictive measure available (CA1, CNG2, CG3, CG5, CG6, PA2, PNG4, PA6). While some participants identified that there was a pervasive *perception* among stakeholders in SIDS that TIAs substantively prevented meaningful nutrition policies from being enacted (CA1, CNG2, PA2), several participants urged that there was ‘actual’ scope for nutrition policy within the confines of trade and investment obligations. Nevertheless, participants noted that the need to make nutrition policies trade-compliant implied some additional burden on SIDS, such as resourcing extra studies and expertise.

…we were working so hard to make sure we had a strong evidence base… So the front of pack labelling studies that were done with the octagonal warning from Chile, [industry] were like, “No, no, no but that works for Chile. But that doesn’t mean it will work in the Caribbean.” And I am like, “Are you kidding me!” But you know what? We went and we did studies… So you make sure you are ticking those boxes. So you say, okay, we make sure we have the evidence. [CA1]

It was noted that SIDS could be vulnerable to litigation if the background work was not completed to a high enough standard to show that the policies were trade-compliant (CA1). Despite this risk, some participants argued that when litigation occurred for breaches of trade obligations, countries that pursued policies for legitimate public health reasons usually prevailed (CA1, PNG4). Participants from both the Caribbean and Pacific often cited the Australia-Philip Morris International case on plain packaging of cigarettes as an example to support this point of view.

However, participants also identified that nutrition policy space was constrained by a lack of understanding by nutrition policymakers that the TIAs substantively allowed for a broad scope of nutrition policies (CA1, CNG2, PA2). Consequently, they stated a belief that certain nutrition policies were not pursued due to the perception by nutrition policymakers that the trade obligations would substantively prevent them from being implemented, despite the rules permitting these.

Vanuatu joined the WTO in 2012 and… it kind of created some regulatory chill in things around policy for health, more because I think there wasn’t great understanding as to what impact it would have. So, people were more conservative in general saying, “We don’t know much about trade so are we still allowed to do things associated with tobacco and associated with soft drinks?” [PA2].

Several participants commented on the impact of the food industry using trade-related arguments to push back against proposed nutrition policies (CA1, CNG2, CNG4, CG5, PNG4). Despite participants agreeing that much of the suggested nutrition policies would have been ‘legally acceptable’, industry used the threat of litigation, relying on a lack of trade expertise among nutrition policymakers, to promote their interests. An example from an experience with the tobacco industry was provided as emblematic of unhealthy commodity industries’ playbook:

I can send you this letter written by British American Tobacco to the PNG [Papua New Guinea] government. If you look at it, they just copied what they did to the government of Uganda on a letter head and changed the date. It was basically the same thing. They threatened them with this provision of the WTO applies when it doesn’t… their interpretation is all incorrect in law. [PNG4]

#### Procedural constraints

Procedural constraints limit the policy making process, placing practical barriers that must be overcome for nutrition policy to be enacted (22). Several participants noted the threat of litigation from Transnational Corporations (TNCs) and other states as a reason specific nutrition policies were not pursued, demonstrating regulatory chill (CA1, CG3, PA2, PNG4, PA6). In some instances, potential legal threats were considered by health and trade policymakers before the development of any nutrition policy and worked to reduce political will in nutrition regulation (CA1, PA2).

The threat of litigation? Yeah, without a doubt. Realistically, I don’t think anybody can dispute that. Anybody who is threatened with litigation, you are going to think twice, and you are going to think twice again, and think, “Do I really, really want to do this? And, am I really convinced that this is the right way to go?” Because litigation is costly. It is time-consuming. [CA1]

One participant used the example of Fiji and New Zealand relations to emphasise the impact of legal threats on constraining nutrition policy space. In this case, Fiji’s mutton flap ban resulted in backlash from New Zealand, threatening the integrity of the policy.

I think it was under the labour coalition government in early 2000 in Fiji… where the then-prime minister, Mahendra Chaudhry, wanted to ban Mutton flaps and there was a huge uproar with the New Zealand government who said it was contrary to WTO rules. Along the similar lines of the turkey tails in Samoa. [PNG4]

Participants commented that industry trade-related arguments were particularly successful where nutrition policymakers lacked the expertise to counter the industry position and required trade experts to spend time and energy to respond to their threats (CA1, CNG4). They also noted that during policy discussions, industry interests “always had a trade expert there” (CNG4). Participants used the example of the front-of-package-label (FOPL) warnings in the Caribbean to demonstrate how industry trade-related arguments had slowed the policy making process.

You counter the argument in one set of consultations, and you think okay that is done and dusted and then the next round of consultations it is back at you and it is the same thing again. And someone is entertaining it and you are just exhausted by the end of it because you are just repeating the same thing over and over again. [CNG4]

#### Structural constraints

Structural constraints in this paper refer to all potential contextual factors in which the trade and investment landscape may limit nutrition policy space. Participants noted that, instead of TIAs being the root cause of trade and investment-related nutrition policy constraints, they may instead be a symptom of a more profound trade liberalisation ideology favouring trade-related economic growth and development imperatives over public health concerns. Nearly all participants noted that health was given a lower policy priority than trade, and when there was tension between the two, trade and economic imperatives dominated the internal policy process (CNG2, CG3, CNG4, PA1, PA2, PA3, PNG4, PG5, PA6). Similarly, even where the policy was clearly a public health measure, trade actors had significant political power limiting nutrition policy space.

…when you talk about food and nutrition and when you talk about trade and investment, food and nutrition are like the poor cousins in the room… It is so hard to be taken seriously when compared to trade and investment. [PG5]

SIDS policymakers were noted by Pacific participants to prioritise trade and the economy in policy decisions due to pressures for SIDS to meet their development and economic goals (PA2, PA3, PG5, PA6). Meanwhile, others noted that trade and health were not necessarily mutually exclusive despite often being perceived this way by SIDS policy stakeholders (CA1, PA6).

I think that the trade obligations are very much seen as this thing we need to do as a product of our development. What it does to the population is seen as almost secondary. [PA2]

Several participants noted the impact of global pressures to join the global trading network and, through working with global partners, SIDS were influenced to adopt trade liberalisation policies and prioritise economic growth (PA1, PA2, PNG4, PG5, PA6).

We are always up against the economic arguments that we need trade, and we need to liberalise, and without it, small island countries like us in the region, we cannot function. [PG5]

One participant noted that, ultimately, nutrition policy space in SIDS has been influenced by the prevailing global neoliberal ideology. Within the context of the international trade liberalisation ecosystem, it was difficult to know whether constraints on nutrition policy space resulted from TIAs themselves or from the “neoliberal economic policy paradigm” (PA6) internalised by SIDS governments.

So, you know, that sort of paradigmatic dimension means that, to go back to what I said initially, disentangling. Is that a free trade agreement or is that a deeper paradigmatic influence on policy, and what is deemed acceptable, particularly for these policies that step outside of the traditional remit of the health sector? [PA6]

Similarly, several participants identified the idea of unhealthy foods as presenting substantial challenges for trade-related regulations (CG3, PA2, PNG4, PA6). They compared the regulation of unhealthy foods with tobacco regulations, noting a greater difficulty in regulating unhealthy foods due to ambiguity among nutrition standards and perceptions of what is ‘unhealthy’. While tobacco was always perceived to be harmful, the healthfulness of food products was thought to change depending on the context in which it was being considered, making nutrition policy making particularly difficult.

But differentiating foods is just fundamentally quite challenging… Is my raisin toast less healthy than wholemeal toast? …I am trained in nutrition and don’t say that lightly. I think it is quite possible to identify healthy foods and unhealthy foods but when you are working with food as a trade commodity the basis for those differences can become quite arbitrary. [PA6]

One participant blamed the food industry for the perception that unhealthy foods were somehow different from other unhealthy commodities (PNG4). They noted that unhealthy and processed foods have become accepted as part of a regular diet through persistent lobbying and marketing from the food industries. The perception of unhealthy foods as different from other unhealthy commodities worked to reduce the nutrition policy space of SIDS.

But I think it is because processed food has been so socialised. We have been conditioned that it is just like any other food when in fact it is not. And it has happened systematically over the years. [PNG4]

Almost all participants noted the impact of the food industry in constraining nutrition policy space (CA1, CNG2, CG3, CNG4, CG5, CG5, PA1, PA2, PA3, PNG4, PA6). Private interest groups were noted to have access to policymakers that civil society actors did not have. Industry used its influence to continuously push back against healthy nutrition policies, which it saw as undermining its bottom line through well-organised and coordinated lobbying. Their influence impacted the local, regional and global political trade and nutrition landscape resulting in structural constraints on nutrition policy space for SIDS.

Their lobbying groups are incredible. It is like this giant international apparatus that goes into full operation when need be. The United Sates is leading the way and you see it in some of the discussions, especially at the World Health Assembly where the US questions rules regarding breast milk substitutes and you know that is the industry talking. [PNG4]

Many Caribbean respondents noted the impact industry had in preventing the proposed regional FOPL system from being legislated (CA1, CNG2, CNG4, CG5). They mentioned how industry was involved in delay tactics such as requesting more evidence before initiating legislation, lobbying politicians to vote against nutrition regulations, funding and conducting research favouring their interests, supporting less effective alternative regulations and shifting the narrative from population interventions to individual responsibility. One participant considered the pressure from industry to be the most significant problem facing nutrition policy space in the Pacific and the Caribbean.

That is why to my mind a lot of what happens in the public health space is not about trade. It is not really about trade. It is about industry interference… we have a whole unhealthy food industry that is about making money from selling unhealthy food. And anything that you are going to do that is going to negatively impact [industry interests], there is going to be pushback. [CA1]

### Challenges faced by SIDS

#### Financial and capacity constraints

Most participants noted that SIDS have been limited in their financial means and available workforce capacity to undertake the required tasks of maintaining nutrition policy space in the face of trade and investment constraints (CA1, CNG2, CNG4, CG5, CG5, PA1, PA2, PG5, PA6). They discussed that, as many are low- and lower-middle-income states, the development imperative has been critical for SIDS to ensure their economic progress. Where there was tension between SIDS’ immediate economic survival and the population’s long-term health, resources have been dedicated to supporting economic initiatives, thereby limiting resources available for maintaining nutrition policy space.

When you talk about small island developing states, given our already remoteness and insularity and all those characteristics that make up small island developing states, it is very hard to talk about food and nutrition without asking about the dollar value or the compromise you are wanting to make with trade and investment. [PG5]

Similarly, the location and delicate ecosystems of SIDS meant that they faced frequent natural disasters such as hurricanes, placing a greater resource and capacity burden on governments (CG5, PA2). Participants noted that these emergencies regularly diverted resources away from supporting nutrition policy space.

… a lot of what is happening in the Pacific in health is putting out spot fires. There is always an outbreak of this or some kind of disease or response to emergency so we don’t have time to sit and think strategically about how we can take some pressure off the system in the long run by having fewer people with NCDs early in life. [PA2]

Participants also noted that SIDS’ small populations made it difficult for them to have personnel with expertise in the niche areas of nutrition and trade policy. Experts were necessary to combat food and beverage industry trade-related arguments that push against nutrition policies, to construct trade-compliant nutrition policies, and ward against regulatory chill. Participants stated that formulating the evidence base for nutrition policy was also a resource-intensive initiative and was particularly difficult for SIDS to undertake due to financial constraints. Participants expressed that given their small size, it was unrealistic to expect every SIDS to have an expert in every policy domain, and inevitably there is limited capacity for nutrition and trade experts.

Despite SIDS’ capacity limitations, some participants also noted that key actors successfully maintained nutrition policy space. While there may be few, there are trade and nutrition experts who play important roles and are *“brilliant at their jobs”* (PA2) in the Pacific and Caribbean. In the Caribbean, for example, one key actor had been an active advocate for nutrition policy and was the main person responsible for providing advice to counter trade-related industry arguments relating to the regional FOPL policy (CNG4). However, the sparsity of these essential actors put SIDS’ nutrition policy space at risk if they were to leave.

#### Greater industry influence and conflict of interest (COI)

The smallness of SIDS placed them at particular risk of industry regulatory capture, constraining their nutrition policy options. All participants noted the industry’s strong influence in pushing back against healthy nutrition regulatory policies, and several expressed how this was a particular problem for SIDS (CA1, CNG2, CNG4, CG5, PA2, PA3, PNG4, PA6).

I think for the Caribbean, because our region is small, the countries are small and therefore the capacity or the space for significant interference and influence of our policymakers systems is very, very strong. [CNG2]

Industry actors were noted to have significant political sway in several SIDS. Unhealthy commodity industry actors were said to have achieved positions of influence due to their coordination, amplifying their influence (CA1, CNG4, CG5).

I think there is also other risk sometimes in small economies of regulatory capture, of very dominant players which are quite influential politically and otherwise, perhaps having way more influence than groups like civil society for instance. [CG5]

Several participants perceived that much of the industry influence in SIDS stemmed from the fact that actors wore “many hats” and often played multiple, sometimes competing roles in government and industry positions (CA1, CNG4, PA1, PA2, PNG4, PA6). This led to potential conflicts of interest in the policy making process, constraining the scope of healthy nutrition policies. The extent to which industry and political interests overlap was thought to give industry actors greater access to politicians and nutrition policy decision-making than in other larger, more economically developed countries.

When you are dealing with small island states, you have people wearing multiple hats. You don’t have the luxury of having this clear separation of this person is the bad industry and this person is the good industry, because sometimes you even have the same man as the manufacturer and even doing health products and non-health products or doing pharmaceuticals and also doing unhealthy food. [CA1]

#### Limited international power

SIDS were thought to have limited international power compared to other international trade and investment players, impacting their ability to negotiate favourable TIA terms (CNG4, PA1, PA2, PNG4). Several participants highlighted the imbalance of power in the trade negotiations, particularly in the Pacific Region, with the “heavy-handed” role of Australia and New Zealand in ensuring the terms of the TIAs were in their interests rather than the interests of SIDS.

You know when Vanuatu sits down with Australia and New Zealand, they are going to sit down with a team of 30 lawyers, you know, with a Vanuatu person… It is such an imbalance of power when you negotiate. It is so hard. [PA1]

The imbalance of power in trade negotiations resulted in TIAs that did not reflect the development interests of SIDS. Participants described the resultant TIAs as allowing the increased flow of harmful commodities into SIDS while failing to significantly safeguard nutrition policy space or provide other discernible benefits to offset their health costs (PA1, PA2, PA6).

In trade agreements small parties always miss out. So, when you are thinking small when you get to Pacific Islands that is as small as you get… Small island states aren’t large exporters. They import more than they export so how can these things… Opening up and reducing the amount that you can claim on things, how is that ever going to be in your interest? [PA2]

One participant used the example of the PACER-plus negotiations between Australia and Vanuatu to show how Australia leveraged the already established labour scheme that Vanuatu relied on for seasonal work in Australia to encourage Vanuatu’s participation in PACER-plus (PA1). The participant claimed that Vanuatu would not have signed into the PACER-plus agreement if Australia had not packaged it with the ongoing running of the labour scheme, stating, *“that was the reason why it ended up being signed the way it was.”* (PA1).

### Solutions for improving nutrition policy space

#### Intersectoral capacity building

One of the participants’ leading solutions for improving nutrition policy space in SIDS was to build SIDS’ capacity to formulate robust nutrition policy within the current trade landscape (CA1, CNG2, CNG4, CG5, PA1, PNG4, PA6). This was suggested to involve training local personnel to better develop trade-compliant nutrition policy, counter the trade-related arguments brought forward by industry, and strengthen SIDS’ position within trade negotiations so that the potential impacts on health were identified and addressed before any TIAs were signed. Participants commented on how training and support had helped maintain their nutrition policy options in the face of trade-related arguments.

You know Nestle comes in and they bring… they don’t trust your local lawyers, so they bring in their entourage, their executives with suits and they try and intimidate us but you know, thanks to the knowledge that we gained from different workshops in talking to the experts they are very confident to deal with them. [PNG4]

Building a workforce that has the understanding and political will to commit to potentially challenging nutrition policy was viewed by some participants as essential to supporting nutrition policy space (CNG4, CG5, PA1, PNG4).

And that is why to my mind the two critical things in this process when we are looking to implement public health policies that are effective and can withstand scrutiny and challenge. The two things are critical, the evidence, making sure that we do things in the right way. You know, following the process that we are to go through. But also linked to that then is, you need to have people that understand the trade rules. [CA1]

Ensuring that trade expertise and nutrition policy advocacy are maintained into the future was identified as an essential component of capacity building. One participant noted the significance of developing institutions that outlasted any particular individual, given the rapid turnover of officials in SIDS and difficulties with political will (CG5). They noted that nutrition policy could take time to overcome the procedural and structural constraints placed by trade and investment and therefore required ongoing support from nutrition advocates knowledgeable in trade law.

It is a continuous battle so you need to maintain that kind of momentum and it is best done by developing that cadre of personnel across the region who will continue the efforts. There are some efforts to do so. The Healthy Caribbean Coalition have some youth organisations built around it that takes up the mantle of NCDs and promoting it around their colleges in schools and so on. [CG5]

Similarly, intersectoral collaboration was identified by some participants as being essential to maintaining nutrition policy space against TIA constraints (CNG4, CG5, CG5, PNG4, PG5, PA6). Many participants noted the importance of having health advocates in trade discussions and decision-making to ensure that nutrition interests were maintained within TIAs, while others identified the importance of having trade expertise in nutrition policy development to ensure that nutrition policies were safe from litigation.

The officials, the problem is, trade is under the gamut of specific government ministries. And governments, they keep talking about multisectoral collaboration but it is almost out of reach because they don’t practice it. And to me that is essential. If you want to get a broad perspective because every ministry, while their mandates may be different and the path to the mandate is different, the goal is still the same. And I think that issue of multisectoral collaboration is really key. [PNG4]

One participant also suggested shifting reporting so that the ministries in charge of trade were required to report on health and nutrition aspects to align the fundamental drivers behind trade decisions with health and development goals (PA6).

So I guess a strategy for us is to get the dialogue going and to get these different reps from different sectors in the same room just to have an understanding of the issues across the floor. Unless you have an understanding of how things are interconnected you wouldn’t make an effort to come together and to speak about these things. [PG5]

Meanwhile, other participants recognised the inherent constraints of building local capacity in SIDS (CA1, CNG2, CNG4, PA6). SIDS may be small and financially constrained, making building capacity in nutrition and trade impractical when there may be other pressing demands on already stretched government resources. Instead, some participants suggested that SIDS leverage international support to bolster their trade and nutrition capacity (CA1, CNG2, CNG4, CG5, PNG4). These participants gave examples of international support from organisations such as the Pan American Health Organization (PAHO), the McCabe Centre and Bloomberg Philanthropies, which had already assisted in training and technical resources to support nutrition policies facing TIA constraints.

#### Conflict of interest (COI) policies

Several participants suggested COI policies to address the problem of industry interference (CA1, CNG2, PNG4). COI policies institutionalised at the regional level were considered necessary to insulate the policy space from industry capture. While one participant noted the importance of having industry involved in forming policies that might affect their businesses (PNG4), others viewed industry as a powerful constraint on nutrition policy space and COI policies as a means of reclaiming their policy making autonomy.

. the issue of conflict of interest has to become a vital check mechanism and institutionalised within our organisations, within our government institutions. [CNG2]

#### Improve TIAs and the TIA negotiation process

To counteract the significant power gradient between SIDS and larger, more affluent countries when negotiating TIAs, participants suggested improving the trade negotiation process and renegotiating the current stock of unfair TIAs (CNG2, CG5, PA1, PNG4). One participant used the example of Vanuatu’s negotiations with Australia over PACER-Plus to suggest that SIDS could build capacity in the trade negotiation process with more expertise and personnel to level the playing field.

You could bring in people who are better at trade negotiations, like you could train them on trade negotiations. You could try to have like 5 people sitting for the Vanuatu side when they have a meeting with 30 people from Australia. [PA1]

Others suggested involving health experts in the TIA negotiation process so that health interests were explicitly considered. Similarly, they observed there needed to be better processes that allowed for broader deliberation when negotiating and agreeing on TIAs so that the health consequences of the agreements could be understood before they were ratified.

I know that at the HCC [Healthy Caribbean Coalition] level one of the things that we have talked about is inserting them [health experts] at some of the world trade policy bodies. [CNG2]

One participant suggested that the current batch of TIAs should be renegotiated through improved processes to better reflect the interests of SIDS. By approaching TIAs through the lens of healthy nutrition policy objectives, they suggested that future TIAs could, in fact, be harnessed to further SIDS development goals.

There is so much discourse right now on how investment law, international investment law, can be reimagined, how it can be refashioned in aid or in furtherance of sustainable development objectives. Many people are talking about ripping up the existing rule book and not being constrained by the current paradigm about how investment policy can be used… if you are interested in a certain quality of investment or a certain character of investment, how would you fashion obligations that would send that signal? There could be ways in which your investment policy, not just your investment treaties, but your investment policy can send that clear signal about what kinds of investment or what quality of investment you are interested in. [CG5]

#### Global consensus on public health nutrition

Building a global consensus on public health nutrition measures was suggested by participants to shift international ideology away from promoting trade liberalisation and private industry as the most important drivers of SIDS development (CNG2, PA1, PA2, PA6). Instead, international agreement could support a paradigm that promoted health, nutrition and wellbeing policies alongside economic pursuits.

I definitely have a problem with a focus on narrow economic metrics as somehow signifying success in the development agenda… that interplay between industry and government is not all industry dictating, it is also government going, “Okay guys. Pick up your game. You are spearheading our development agenda.” So that sort of paradigmatic dimension needs disentangling. [PA6]

To change the international ideology, two participants identified that nutrition policy advocates could gain some traction in the trade-dominated policy landscape by framing the debate around nutrition policy space in terms of human rights issues and linking nutrition policies to the 2015 Sustainable Development Goals (CNG2, PA6). For example, one participant noted that bringing the Caribbean Court of Justice to support the Caribbean FOPL policy as a human rights issue to further support their case had been considered (CNG2). By leveraging the framing of human rights and sustainability, these participants believed that SIDS could combat neoliberal ideology and gain greater nutrition policy space by increasing the prioritisation of nutrition policies over trade obligations.

Two others suggested that the nutrition policy debate be framed in economic terms to resonate with economic and trade actors (PA1, PA2). It was noted that health professionals sometimes struggled to communicate the importance of nutrition policy measures in trade debates and couching the argument in economic language was a possible way to reach a larger audience (PA2).

Yes it feels uncomfortable but let’s talk about these things in economic terms or let’s talk about them in environmental terms. We still often use the same language around health and health burden that is not well understood by those we are talking to. It makes for ineffective communication for what matters and how to kind of mobilise people to see things the way we do. [PA2]

Additionally, participants suggested that there needed to be international standards, frameworks and laws that enshrined the pursuit of health and protected nutrition policy space from trade constraints (CA1, CNG2, PA1). Some suggested that world trade bodies should take a more proactive approach to protect domestic nutrition policies by clarifying the ranking of nutrition policies over trade. Others used the example of the Framework Convention on Tobacco Control (FCTC) as a type of international framework that could be adopted for nutrition to support SIDS in their nutrition policy making to reduce the harms of nutrition-related NCDs.

It is so difficult because we have for example FCTC 5.3 that sets out in a treaty… I mean you have settled in a treaty. You can’t get better than that! It is legal language that is explicit… yet you don’t even have a treaty as is the case with food. [CA1]

## Discussion

This research set out to understand the impact of TIAs on nutrition policy space and identify solutions to expand nutrition policy space for SIDS. Using critical realism and grounded theory principles, themes from interviews with key stakeholders in nutrition and trade policy from the Caribbean and the Pacific were identified. Generative mechanisms were identified to describe the TIA constraints placed on SIDS’ nutrition policy space, the associated challenges faced by SIDS and potential solutions for improving nutrition policy space.

While TIAs may not significantly substantively constrain nutrition policy space, they may impose procedural constraints in the form of mitigating or responding to litigation threats and increasing the burden on SIDS policy makers of making nutrition policies trade-compliant. For a nutrition policy to be accepted within the international trade regime, it must be designed in a way that addresses a legitimate public health objective, is evidenced-based, non-discriminatory, non-arbitrary, and necessary, and be the least trade-restrictive measure to achieve the policy goal. Increasingly, governments are compelled to justify and defend their nutrition policies along these lines, in response to intense scrutiny and adverse legal interpretations, from those with opposing economic interests [32, 33]. This can pose significant challenges for any government, let alone small, isolated economies with resource constraints. Regulatory chill from TIAs has similarly been well documented as inhibiting policy space in other scholarship in similar policy domains [14, 16, 22, 26].

Consistent with the existing literature [22, 34–37], these procedural restrictions were compounded by the structural constraints that form the institutional and ideological bedrock of the international trade regime and include perceptions surrounding unhealthy foods, trade liberalisation ideology and industry interference. These TIAs have largely been developed within a global neoliberal paradigm, which prioritises “free markets,” deregulation, and limited State intervention, and can be considered a symptom of a deeper ideological worldview prioritising economic development and corporate profit over population health and wellbeing [38]. Previous literature on the commercial determinants of health has posited that powerful economic operators have leveraged neoliberal and capitalist ideologies alongside globalisation and trade liberalisation to increase their sphere of influence and have used TIAs to facilitate the penetration of neoliberal values into developing states, granting greater power to industry interests [18–20]. As documented by Hoe et al., ultimately, industry have been privileged with high levels of influence over nutrition policy processes and have weaponised TIAs to further their interests [39]. The findings from this research reiterate the comments from Milsom et al. [40] that a power shift is needed along with a reprioritisation of public health interests to expand nutrition policy space, particularly for SIDS, which face additional contextual challenges in nutrition policy making.

Our findings suggest that in SIDS, where the burden of nutrition-related diseases is high and international power can be limited, more needs to be done to safeguard nutrition policy space from these TIA constraints. For local actors wishing to expand nutrition policy space, this research can give them confidence that many progressive/effective nutrition policies can be pursued within the current substantive bounds of TIA policy space. However, this research also adds to the public health advocacy literature [41, 42] by recommending building trade and nutrition expertise and capacity through training local personnel and leveraging international connections, fostering multisectoral collaboration to pursue governmental policy coherence and establishing conflict of interest policies to push back against the procedural and structural constraints. This research recognises the constraints placed by the global trade liberalisation ideology and power imbalances in TIA negotiations and aligns with previous work highlighting the need for international collaboration to provide SIDS with capacity assistance to balance negotiating power and increase transparency [21]. Other researchers have also noted the power imbalance small nations face in international trade relations and suggest further investments in negotiating, analysis and strategy [43–46]. Our research extends these recommendations by calling for action from international organisations such as the WTO and WHO to explicitly prioritise nutrition policies to help shift the international trade liberalisation ideology by establishing pro-nutrition policy frameworks and standards, and reprioritise healthy nutrition in the global discourse [47].

One strategy to strengthen nutrition policy space has been to ensure nutrition is safeguarded by writing public health carveouts into TIAs. However, while the EU-CARIFORUM TIA has a clause specifically allowing states to “protect public health and nutrition,” most TIAs do not include nutrition-specific carveouts (49,50). Leaving the interpretation of ‘public health’ up to the trade governing bodies increases the risk of litigation and regulatory chill where opposing parties may interpret policies such as FOPL as falling outside the current ‘public health’ exceptions and places the burden of proof on public health advocates [49, 50]. Alternatively, including clauses that protect the right to regulate or place obligations on trade parties to maintain health standards could potentially encourage stronger public health nutrition policy in SIDS [51].

SIDS rely on trade relationships for economic development and food imports [52]. This research identified a pervasive perception among policy makers in SIDS that economic imperatives and public health are part of a zero-sum game where trade and investment take priority. Alongside TIAs, the prioritisation of policies considered to be economically beneficial while ignoring the implications for health, is another symptom of global neoliberal ideology and places internal barriers for policymakers to implement healthy nutrition policy [53, 54]. However, these two positions are not mutually exclusive, and recent examples have demonstrated that countries can pursue economic and health outcomes in parallel. For example, Brazil has shown how investment agreements can be forged to promote the country’s wellbeing interests through Cooperation and Facilitation Investment Agreements [55]. As these agreements contain investment facilitation rather than protections, they increase states’ regulatory space in areas such as health, labour and the environment and place corporate social responsibility obligations on investors. As Delany et al. [50] have argued, improving the process of TIA negotiations and aligning trade and nutrition goals through improved agreements, improving dispute-resolution processes, deferring to health, environmental and human rights frameworks, limiting investor privilege and enforcing corporate responsibility can make TIAs work in the interests of SIDS.

### Strengths and limitations

The strengths of this research lie in its in-depth exploration of stakeholder experiences and views regarding TIAs and nutrition policy space in SIDS, guided by established frameworks and theories. It is the first to explore the impact of TIAs on nutrition policy space and solutions for expanding nutrition policy space specifically for SIDS. SIDS, arguably, are some countries with the least international trade power and the greatest need for substantial population nutrition policies; therefore, research in this area is of particular value.

However, a key limitation was the requirement for selected participants to be able to provide an interview in English, and there is likely bias towards the experiences of English-speaking experts from English-speaking countries. Similarly, this research necessarily involved generalising the experiences of SIDS when, in reality, they all function in unique social, cultural, political, economic and geographical contexts. As a result, many SIDS voices could not be included due to the number of SIDS and practical limitations on sample size. However, the coherence of experiences, ideas and views within each region and between the Pacific and Caribbean increases the generalisability of these results for other SIDS, both within these regions and abroad.

## Conclusion

This research demonstrated that while TIA obligations were unlikely to substantively prevent meaningful public health nutrition policies if designed to meet trade requirements, TIAs may place procedural and structural constraints that risk preventing, postponing or diluting potential nutrition policies. These constraints may be particularly problematic for SIDS due to several contextual challenges. Despite this, local, regional and international actors can increase the nutrition policy space of SIDS through capacity building, fostering multisectoral collaboration, developing conflict of interest policies, improving the TIA negotiation process, and championing the prioritisation of public health nutrition.

## Data Availability

No datasets were generated or analysed during the current study.
